# Supplementary material for: Consumption of an Oil Palm Fruit Extract Promotes Large Bowel Health in Rats
Source: Nutrients. 2020 Feb 28;12(3):644. doi: 10.3390/nu12030644 (PMC7146302; doi:10.3390/nu12030644)

Supplementary Figure S1. Colonic tissues were prepared and stained for histochemical analysis enabling the measurement of the effects of treatments on colonic cell wall thickness, crypt length and numbers of goblet cells. The figures show a) an example of colonic wall thickness measures, b) a representative image of a section showing a crypt length (line) and goblets cells (arrows), and example images of stained colonic sections from the c) western diet, d) OPP-F, e) OPP-D, f) HAMS , and g) green tea treatment groups

a) **Example of colonic wall thickness measurements**

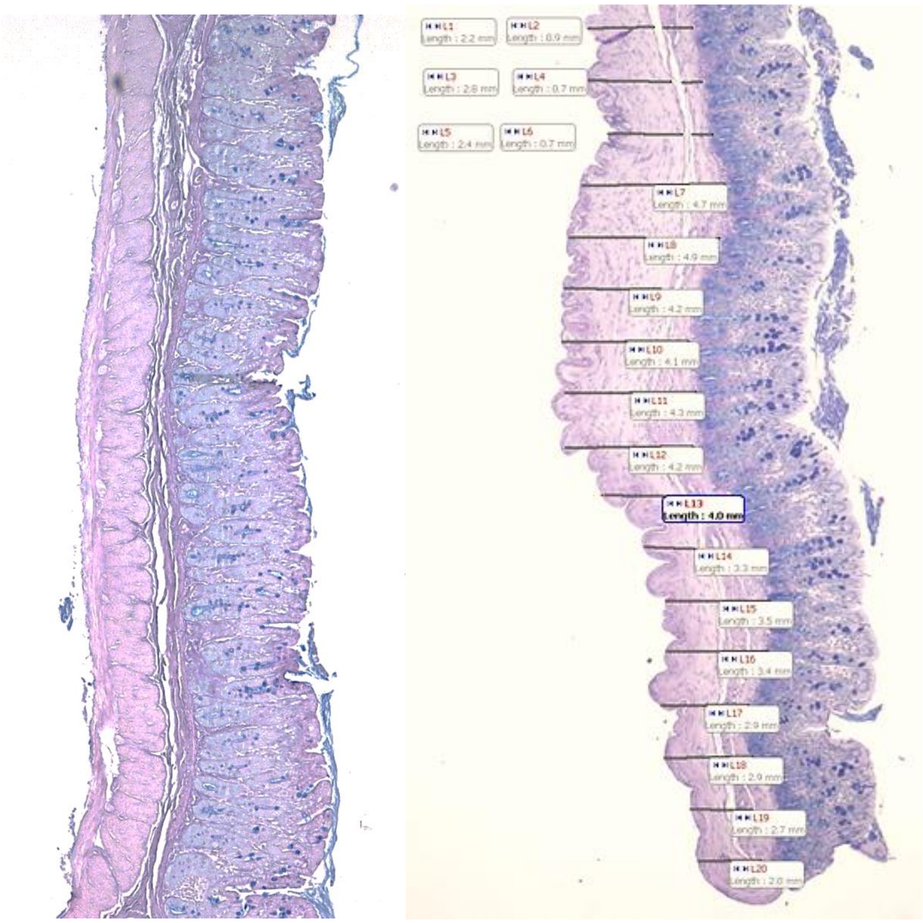

b) Representative marked image showing crypt length and goblet cells

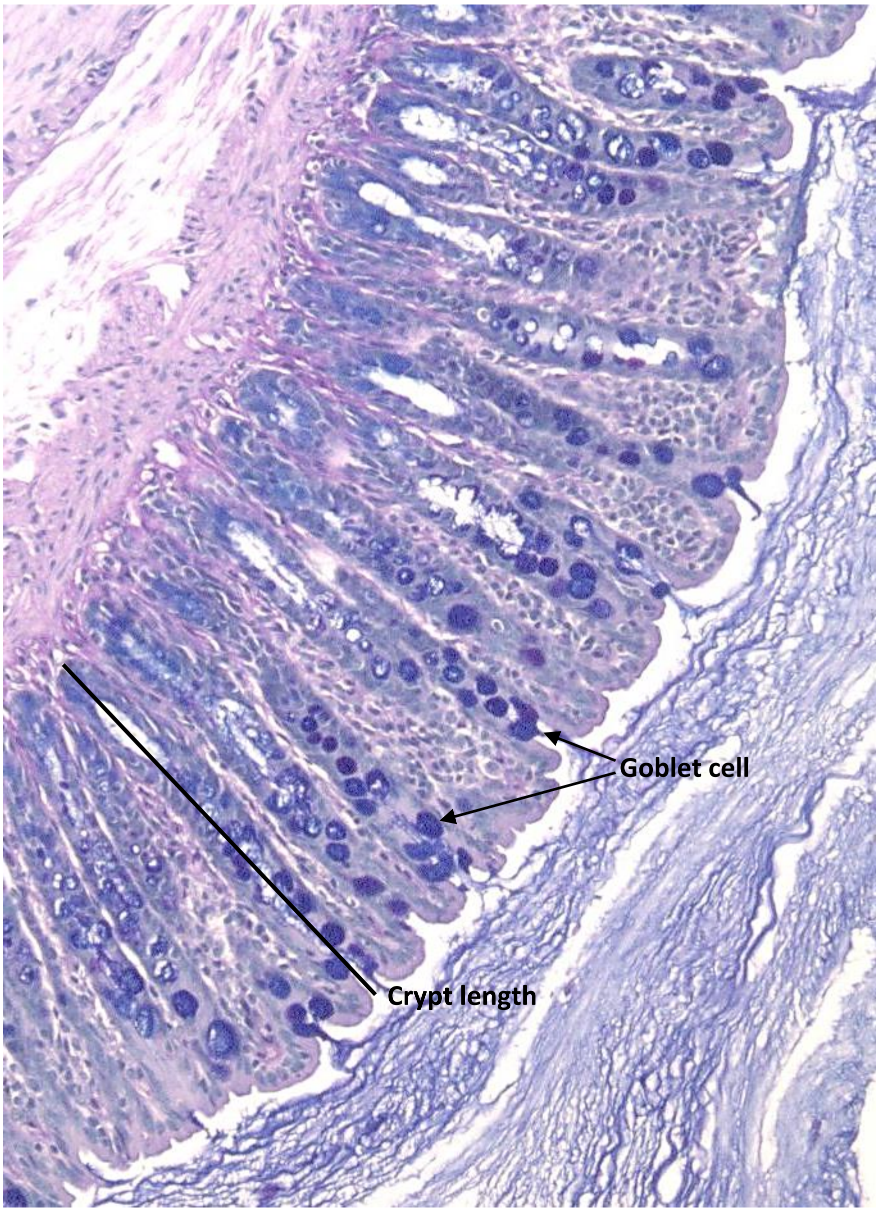

c) Western Diet

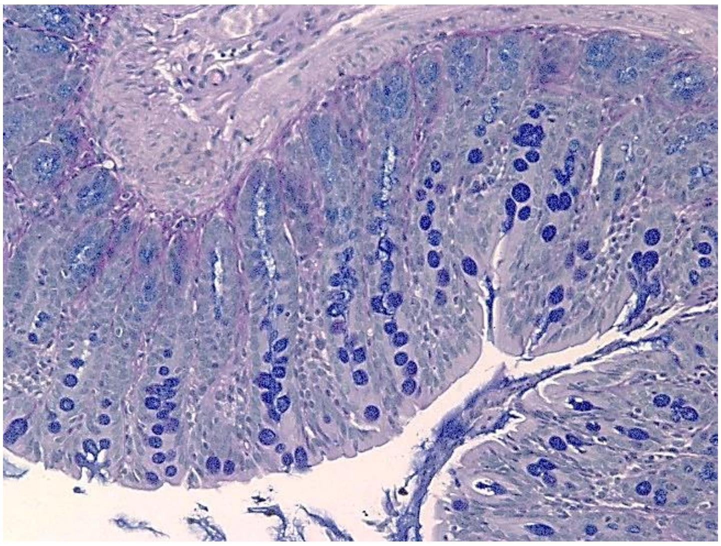

d) OPP-F

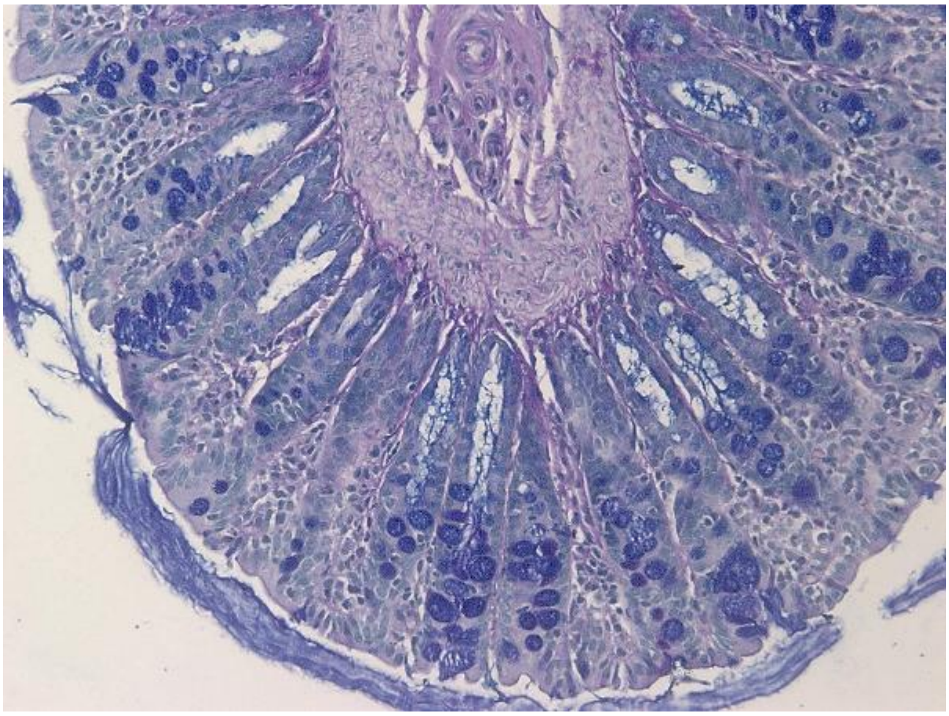

e) **OPP-D**

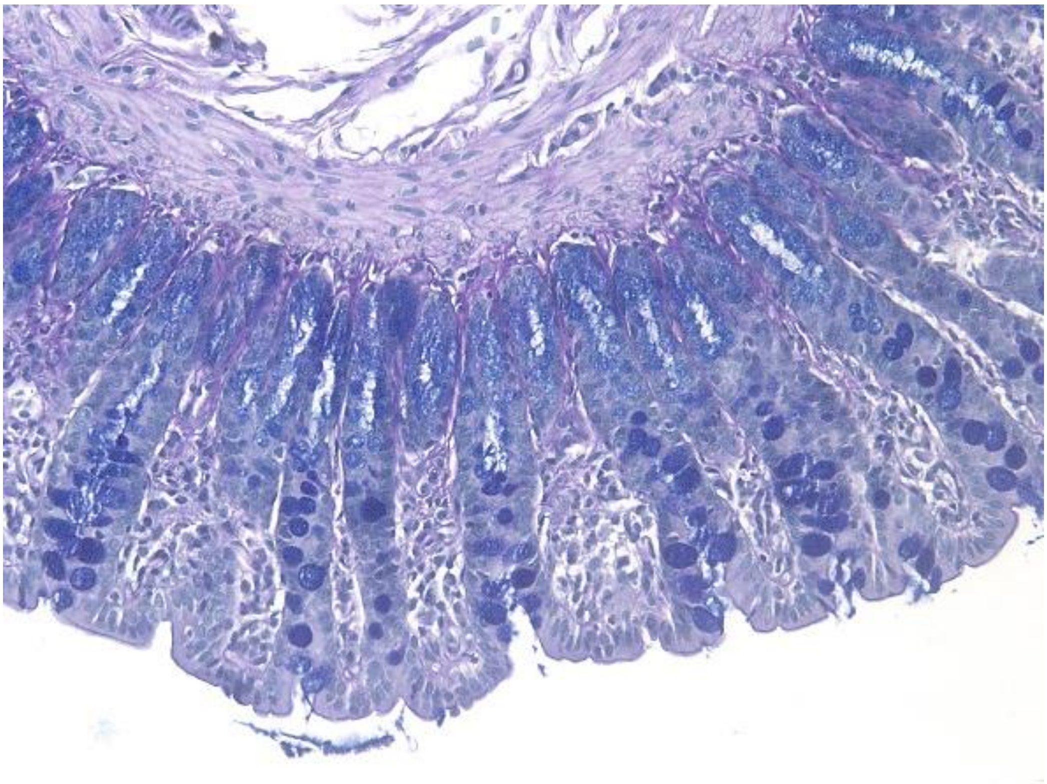

f) **HAMS**

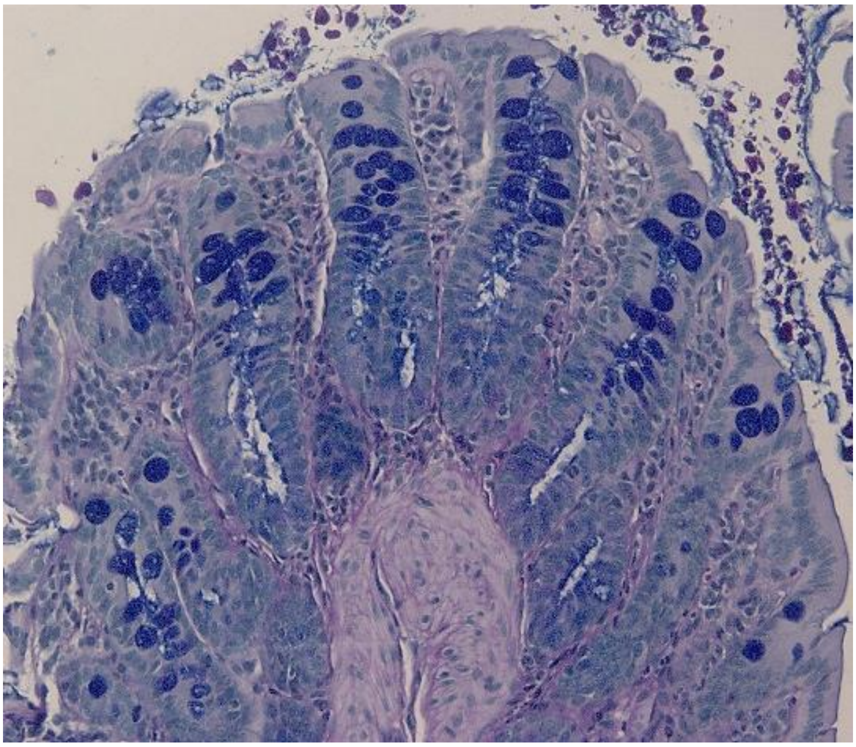

g) Green tea

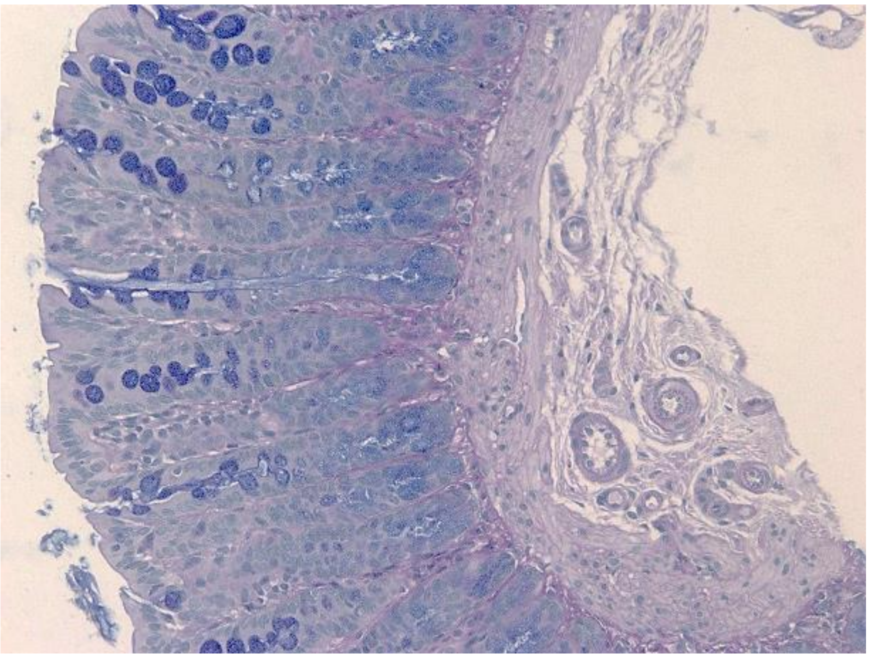

Supplement: Supplementary file 1 [file nutrients-12-00644-s001.pdf]
